# Development of high cell density *Limosilactobacillus reuteri* KUB-AC5 for cell factory using oxidative stress reduction approach

**DOI:** 10.1186/s12934-023-02076-4

**Published:** 2023-04-29

**Authors:** Nisit Watthanasakphuban, Pimsiriya Srila, Phitsanu Pinmanee, Kamonwan Sompinit, Kittipong Rattanaporn, Clemens Peterbauer

**Affiliations:** 1 Department of Biotechnology, Faculty of Agro-Industry, Kasetsart University, Chatuchak, Bangkok 10900 Thailand; 2 Enzyme Technology Research Team, National Center of Genetic Engineering and Biotechnology (BIOTEC), Pathum Thani, 12120 Thailand; 3 Department of Food Sciences and Technology, BOKU-University of Natural Resources and Life Sciences, 1190 Vienna, Austria; 4 Fermentation Technology Research Center, Department of Biotechnology, Faculty of Agro-Industry, Kasetsart University, Bangkok, 10900 Thailand

**Keywords:** Probiotics expression host, Hydrogen peroxide, Superoxide anion, Oxidative stress, Lactic acid bacteria, Cell factory, *Limosilactobacillus reuteri*, Reactive oxygen species

## Abstract

**Background:**

Expression systems for lactic acid bacteria have been developed for metabolic engineering applications as well as for food-grade recombinant protein production. But the industrial applications of lactic acid bacteria as cell factories have been limited due to low biomass formation resulted in low efficiency of biomanufacturing process. *Limosilactobacillus reuteri* KUB-AC5 is a safe probiotic lactic acid bacterium that has been proven as a gut health enhancer, which could be developed as a mucosal delivery vehicle for vaccines or therapeutic proteins, or as expression host for cell factory applications. Similar to many lactic acid bacteria, its oxygen sensitivity is a key factor that limits cell growth and causes low biomass production. The aim of this study is to overcome the oxidative stress in *L. reuteri* KUB-AC5. Several genes involved in oxidative and anti-oxidative stress were investigated, and strain improvement for higher cell densities despite oxidative stress was performed using genetic engineering.

**Results:**

An *in-silico* study showed that *L. reuteri* KUB-AC5 genome possesses an incomplete respiratory chain lacking four menaquinone biosynthesis genes as well as a complete biosynthesis pathway for the production of the precursor. The presence of an oxygen consuming enzyme, NADH oxidase (*Nox*), leads to high ROS formation in aerobic cultivation, resulting in strong growth reduction to approximately 25% compared to anaerobic cultivation. Recombinant strains expressing the ROS scavenging enzymes Mn-catalase and Mn-superoxide dismutase were successfully constructed using the pSIP expression system. The Mn-catalase and Mn-SOD-expressing strains produced activities of 873 U/ml and 1213 U/ml and could minimize the ROS formation in the cell, resulting in fourfold and sevenfold higher biomass formation, respectively.

**Conclusions:**

Expression of Mn-catalase and Mn-SOD in *L. reuteri* KUB-AC5 successfully reduced oxidative stress and enhanced growth. This finding could be applied for other lactic acid bacteria that are subject to oxidative stress and will be beneficial for applications of lactic acid bacteria for cell factory applications.

**Supplementary Information:**

The online version contains supplementary material available at 10.1186/s12934-023-02076-4.

## Background

The application of lactic acid bacteria (LAB) for the production of functional food, drug and vaccine delivery and as cell factory for protein and industrial chemical production has received increased attention due to their efficiency and their GRAS (Generally Recognized As Safe) status [28, 42]. Many LAB produce antimicrobial agents such as organic acids, bacteriocins, reuterin etc. [48]. Food grade and non-food grade expression systems for LAB have been developed and showed high efficiency for homologous and heterologous recombinant protein expression [17, 24, 31, 38], which is applicable for industrial biotechnology. LAB have been reported to show high tolerance to environmental stress such as acid, osmotic and alcohol stress [42], which is beneficial for cell factory applications under harsh production conditions such as high substrate or product concentration and high acidity.

LAB are facultative anaerobic bacteria and require low or no oxygen for growth (Panagiota, Efterpi, and Bonos 2013). Many LAB are sensitive to oxidative stress and suppress cell growth in an aerobic environment, where oxidative stress has been reported as a critical factor for LAB viability and product quality [16]. NADH oxidase (Nox), pyruvate oxidase (Pox) and lactate oxidase (Lox) are oxygen consuming enzymes founded in LAB that decompose oxygen in the cell [16]. These activities result in the formation of reactive oxygen species (ROS) such as hydrogen peroxide (H_2_O_2_), superoxide anion (O_2_^−^) and hydroxyl radicals (OH^•^), which are highly toxic to the cell [43] and lead to protein inactivation and DNA damage. This oxygen-sensitive characteristic is detrimental for industrial applications especially in large scale bioreactors, where anaerobic conditions are difficult to control. Some LAB have mechanisms to overcome oxidative stress such as *Lactococcus lactis,* which can switch to respiratory metabolism in aerobic condition in the presence of heme [13, 40]. But most lactic acid bacteria lack some components for respiration such as cytochrome oxidase or a complete menaquinone biosynthesis pathway, which requires chorismate as precursor and eight biosynthetic enzymes (MenA-H) [49]. In aerobic bacteria the presence of ROS scavenging enzymes such as catalase, peroxidase and superoxide dismutase minimizes the effect of free radicals [43] but these enzymes as well as the required heme cofactor are rarely found in LAB [13, 16]. Alternatively, manganese-dependent antioxidant enzymes including Mn-pseudocatalase and Mn-SOD have been reported in several LAB species including *Lactiplantibacillus plantarum* and *Lentilactobacillus sakei* [1, 2, 16].

*Limosilactobacillus reuteri* KUB-AC5 is a probiotic lactic acid bacterium isolated from broiler gut, which produces some health-related substances such as an antimicrobial peptide and vitamins [12, 32, 34]. The strain could be developed as a cell factory for food-grade products, but its oxygen sensitive characteristic is a crucial limitation, as it is in many other LAB. This approach aims to analyze and understand the oxidative stress and the defense mechanism in *L. reuteri* KUB-AC5 through an in silico study. Strain improvement was then performed using genetic engineering to minimize oxidative stress and facilitate the formation of higher cell density.

## Materials and methods

### Bacterial strains, plasmids, primers and culture condition

The relevant features of expression plasmids, primers and bacterial strains used in this work are listed in Table 1 and Table 2.

**Table 1:** Bacterial strains and plasmids used for this study

Strains and plasmids	Relevant features	Source
Strains		
* Escherichia coli*		
MC1061	Cloning host *recA*^+^	MoBiTec
*Limosilactobacillus reuteri*		
KUB-AC5	Probiotic lactic acid bacteria	Lab stock
pSIP411 + *kat*	KUB-AC5 carrying catalase gene in pSIP411 expression plasmid	This study
pSIP411 + *sod*	KUB-AC5 carrying superoxide dismutase gene in pSIP411 expression plasmid	This study
Plasmids		
pSIP411*gusA*	*spp*-based expression vector, PSIP401 derivative, *gusA* controlled by P_*sppQ*_ (P_orfX_), *sppKR* expression driven by *eryB* read through, with* SH71rep*	[44, 45]
pSIP411 + *kat*	ery, pSIP401 derivative, Mn-catalase gene for *L. reuteri*, with* SH71rep*	This study
pSIP411 + *sod*	ery, pSIP401 derivative, Mn-superoxide dismutase gene for *L. reuteri*, with* SH71rep*	This study

**Table 2:** Primer used in this study

Primer	Sequence (5′-3′)	Target
pSIP F	AAGCATAATGGTGTTATAGCG	P_orfX_
pSIP R	AGCAACACGTGCTGTAAT	MCS
Kat F	ATACCATGGTTAAACATACTAAGATGTTACAACAT	Mn-catalase
Kat R	GAGGAATTCTTAATGATGATGATGATGATGATATTCAC	Mn-catalase
Sod F	GGTATGGGTCTCCGGCATACGAACTTCCAGA	Mn-SOD
Sod R	GGTATGGGTCTCCATGATGATGATGATGATGGTACT	Mn-SOD

*L. reuteri* KUB-AC5 (1% inoculum) was grown under non-aerated (anaerobic) condition at 37℃ in MRS broth (Difco). *E. coli* MC1061 was used as an intermediate cloning host and was grown at 37 °C in LB medium with agitation at 200 rpm. Solid media contained 1.5% agar. Antibiotics were used at final concentrations of 10 μg/ml erythromycin (ery) for *L. reuteri* strains carrying pSIP plasmids and 400 μg/ml for *E. coli* MC1061 carrying pSIP plasmids.

### Growth profile and oxidative stress analysis of *L*. reuteri KUB-AC5

Effect of aerobic growth of *L. reuteri* KUB-AC5 was determined. Overnight cultures (0.1% v/v) were transferred into 50 ml MRS broth in 250 ml Erlenmeyer flasks and incubated without aeration (static culture) and with aeration using an orbital shaker at 150 rpm for 24 h. At three-hour intervals, samples were taken for OD_600_ nm and pH measurement, and the growth profile of shaken and non-shaken samples were compared. The samples of cell cultures at early stationary phase (15 h) were collected and analyzed for ROS (Cellular ROS assay kit, ab113851) as well as catalase (Sigma, Catalase assay kit) and SOD activity (Sigma, SOD assay kit). Moreover, the cellular ROS assay was performed by collecting cells in a conical tube with a final OD_600_ of 0.5, then washing with PBS buffer and incubating with 20 μM 2’,7’- dichlorofluorescin diacetate (DCFDA) solution at 37 °C for 30 min in the dark. After that, the cell pellets were washed and were resuspended in PBS buffer by maintaining the same cell concentration. Then, the cell suspension was transferred to a 96 well black/clear bottom microplate and immediately measured for fluorescence intensity using excitation wavelength at 485 nm and emission wavelength at 535 nm. The measured DCF fluorescence intensity was reported in RFU, which represents the ROS generation.

### Genomic analysis for respiratory metabolism, oxidative stress and ROS scavenging genes

The relevant genes involved in respiration of lactic acid bacteria (dehydrogenases, cytochrome bd oxidase and menaquinone biosynthesis) were analyzed using the whole-genome sequence of *L. reuteri* KUB-AC5 [20]. The possibility for the construction of complete respiratory chain in *L. reuteri* KUB-AC5 was analyzed.¯ An in-silico study of the previously annotated genes of *L. reuteri* KUB-AC5 were blasted against Uniprot and KEGG database. The genes encoding oxidative stress-related enzymes were analyzed and the strategies for oxidative stress reduction or increasing of oxidative stress tolerance in *L. reuteri* KUB-AC5 were identified.

### Construction of ROS scavenging enzyme expression strains

The nucleotide sequences of two selected genes encoding ROS scavenging enzymes (Mn-*kat*, Mn-*sod*) from *Bacillus subtilis* subsp. *subtilis* 168 were codon optimized and synthesized (GenScript, USA) for expression in *L. reuteri*. The oligonucleotides of Mn-*kat* and Mn-*sod* genes were amplified by polymerase chain reaction (PCR) using the primer Kat F, Kat R and Sod F, Sod R. The PCR products were digested with *Nco*I (5´-end) and *Eco*RI (3´-end) and ligated into sakacin inducible expression vector pSIP411. Ligation mixtures were transformed into *recA* + intermediate cloning host *E. coli* MC1061 competent cells and verified by sequencing with specific primers (Table 2). The correct expression constructs were transformed into three different *L. reuteri* KUB-AC5 competent cells prepared according to Watthanasakphuban et al. [49] and van Pijkeren and Britton [39]. Restriction enzymes and ligases were purchased from New England Biolabs, MA.

### ROS scavenging enzymes expression

The *L. reuteri* KUB-AC5 recombinant strains carrying expression plasmids with genes encoding ROS scavenging enzyme, *kat* and *sod,* were checked for the protein expression. Single colonies of the expression strains were inoculated into 25 ml MRS broth with erythromycin and were cultivated anaerobically at 37℃ until OD_600_ = 0.4–0.6, then induced with inducing peptide (IP-673). Non-induced samples were used as control. After induction, the samples were incubated at 3 different temperatures at 25, 30 and 37 ℃ for 18 h. OD_600_ nm was measured and 10 ml of cell pellets were collected using centrifugation at 4000x*g* for 15 min and washed twice with 100 mM sodium-phosphate buffer.

Cell disruption was done using glass beads with BeadBug^™^ microtube homogenizer (Benchmark Scientific, USA) for 5 passages using speed at 4000 rpm for 1 min. Cell lysates were harvested by centrifugation (25,000x*g*, 1 h, 4 °C). The collected cell lysates were measured for the protein concentration using Bradford assay [6] using bovine serum albumin as a standard protein. The size and protein expression of catalase and superoxide dismutase from recombinant strains were identified by sodium dodecyl sulphate–polyacrylamide gel electrophoresis (SDS-PAGE) and confirmed by Western blot analysis using the Bio-Rad electrophoresis and blotting protocol with some modifications. The cell lysates (30 mg protein) were mixed with Laemmli buffer (1:1) and heated at 99 °C for 3 min before being loaded into Mini-PROTEIN TGX Stain-free precast gel (10%) (Bio-Rad, USA). The protein bands were visualized using a stain-free enable Bio-Rad UV-transilluminator (ChemiDoc XRS + , Bio-Rad, USA). The Western blot was performed by transferring the protein bands on SDS-gel to the membrane using a Trans-blot turbo system (Bio-Rad, USA). After overnight blocking, the membrane was incubated with 9 ml of primary antibody (1.8 µl, 1:5000 dilution) solution (Bio-Rad, USA) follow by 9 ml of conjugated secondary antibody (4.5 µl, 1:2000 dilution) (Bio-Rad, USA). The result was visualized using ChemiDoc Imaging System (Bio-Rad, USA).

### Aerobic growth phenotype of recombinant *L. reuteri* strains

The growth phenotype of *L*. *reuteri* variant strains carrying an inducible plasmid was investigated under aerobic condition, activated cultures were transferred into 50 mL MRS broth containing 10 µg/mL ery in 250 ml Erlenmeyer flasks, and incubated at 30 ℃, 150 rpm agitation speed. 25 ng/mL of Sakacin P induction peptide (SppIP) was added to induced treatments at OD_600_ = 0.2 (~ 3–6 h), and sterile water was added to the non-induced treatments (control). At three-hour intervals, one milliliter of culture samples was taken for the OD_600_ and pH measurement. The cell pellets at 24 h of cultivation were collected for catalase and superoxide dismutase activity analysis. Growth, pH, enzyme activity and ROS of induced and non-induced clones were compared.

## Results

### Growth phenotype of *L*. reuteri KUB-AC5

**Fig. 1:**
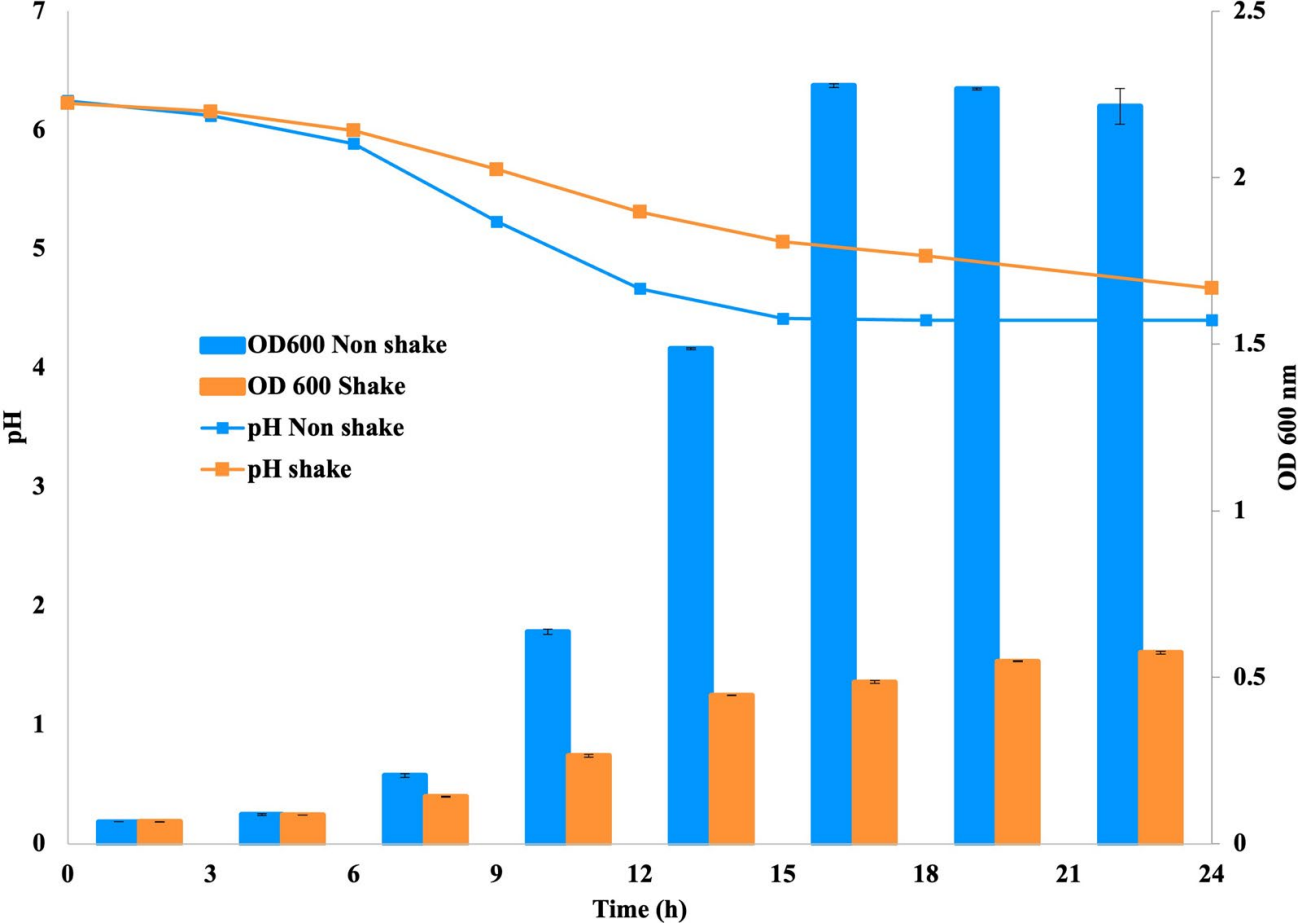
Growth (OD 600 nm) and pH profile of *L. reuteri* KUB-AC5 cultivated in aerated (150 rpm shaking) and non-aerated (non-shake) condition at 30 ℃ for 24 h. All results are the mean of three independent experiments; the error bars indicate the standard deviation

The growth profile of *L. reuteri* KUB-AC5 was investigated under aerobic and anaerobic conditions. In anaerobic condition (without shaking), the OD_600_ of *L. reuteri* KUB-AC5 increased exponentially after 6 h cultivation and reached the stationary growth phase after 15 h cultivation, with a maximum OD_600_ of 2.3 (Fig. 1). Cultivation of *L. reuteri* KUB-AC5 in aerobic conditions with 150 rpm shaking speed did not show a different cell growth (OD_600_) in the early phase of growth (0–3 h). A growth limitation was detected from 6 h of cultivation and was clearly seen after 9 h with an OD_600_ of 0.26 and 0.64 in aerobic and anaerobic cultivation, respectively. OD_600_ of aerated cultures continued to increase slowly after 12 h cultivation, to a value of 0.48 after 15 h and a maximum OD_600_ after 24 h of 0.55, which was 4-times lower than under non-aerated conditions.

The pH profile of *L. reuteri* KUB-AC5 in anaerobic cultivation shows a decline from the initial pH (pH 6.0) until 15 h of cultivation to pH 4.4, after which it remained stable. Under aerobic cultivation, the decline was slower and continued steadily to a final pH of 4.9 (Fig. 1).

### Respiration metabolism, oxidative stress and antioxidant genes

#### Genes involved in respiratory metabolism

**Fig. 2:**
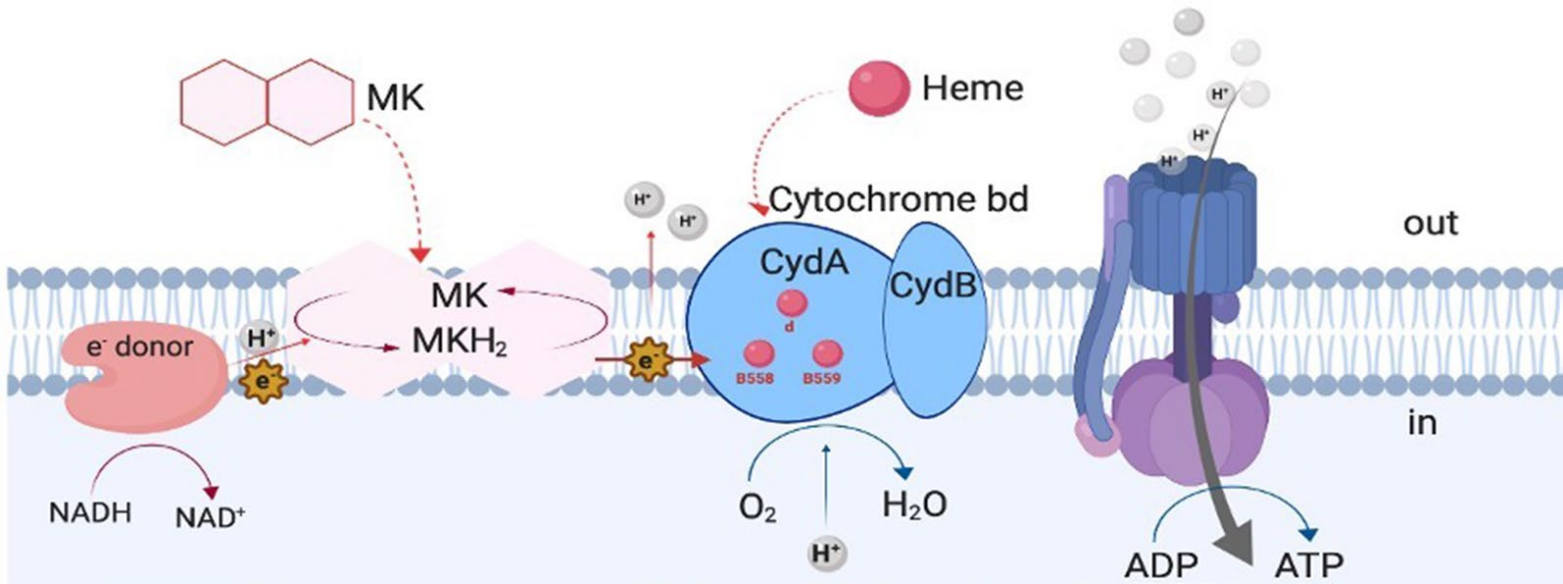
Essential components and mechanism for respiration metabolism in lactic acid bacteria. The LAB respiratory chain comprises an electron donor, an electron carrier (menaquinones) and a terminal electron acceptor (cytochrome bd-oxidase). Heme must be added exogenously to activate the cytochrome bd-oxidase

The complete genome sequence of *L. reuteri* KUB-AC5, which was previously reported [20], was analyzed for the presence of genes encoding components required for respiratory metabolism and compared to the respective complete pathway of *Lactococcus lactis* supsp. *lactis* MG1363 from KEGG database [21]. The entire respiratory pathway in lactic acid bacteria is shown in Fig. 2, including an electro donor (NADH dehydrogenase), electron carrier (menaquinones) and heme-dependent cytochrome bd oxidase as an electron acceptor [7–9, 49]. The orthologous genes of *L. reuteri* KUB-AC5 related to respiratory metabolism are summarized in Table 3.

**Table 3:** Putative enyzmes of *L. reuteri* KUB-AC5 involved in respiratory metabolism and oxidative stress

Gene ID	Protein function	Role in respiration metabolism
Respiratory metabolism		
AC5u0009GL002057, AC5u0009GL000675, AC5u0009GL001904	NADH dehydrogenase	Electron donor
AC5u0009GL000677, AC5u0009GL000678, AC5u0009GL000679, AC5u0009GL000680, AC5u0009GL000676	Menaquinol oxidase	Terminal electron acceptor
AC5u0009GL001766	O-succinylbenzoate-CoA (menE)	Menaquinone biosynthesis enzyme
AC5u0009GL001767	Naphthoate synthase (menB)	Menaquinone biosynthesis enzyme
AC5u0009GL001768	Naphthoyl-CoA hydrolase (menI)	Menaquinone biosynthesis enzyme
AC5u0009GL002179	1,4-dihydroxy-2-naphthoate octaprenyltransferase (menA)	Menaquinone biosynthesis enzyme
AC5u0009GL001463	Demthylmenaquinone methyltransferase (menG)	Menaquinone biosynthesis enzyme
Oxidative stress		
AC5u0009GL000396	NADH oxidase (NOX1)	H_2_O_2_ forming
AC5u0009GL000396	NADH oxidase (NOX2)	H_2_O forming
AC5u0009GL000798, AC5u0009GL001474, AC5u0009GL000835, AC5u0009GL001080	Thioredoxin reductase (TrxR)	Thioredoxin system
AC5u0009GL001491, AC5u0009GL000913, AC5u0009GL001491, AC5u0009GL000913	L-methionine-R-sulfoxide reductase (MsrAB)	Thioredoxin system

The in silico study revealed that the genome of *L. reuteri* KUB-AC5 contains three genes putatively encoding NADH dehydrogenase and five genes putatively encoding cytochrome bd oxidase (Table 3). Several genes encoding enzymes in the menaquinone biosynthesis pathway were found in the genome, namely O-succinylbenzoate-CoA synthetase (menE), naphthoate synthase (menB), naphthoyl-CoA hydrolase (menI), 1,4-dihydroxy-2-naphthoate octaprenyltransferase (menA), and dimethylmenaquinone methyltransferase (menG) (Table 3). Genes encoding the enzymes for the first four steps of menaquinone biosynthesis (menF, menD, menH and menC) are missing. Moreover, genes encoding the pathway for the formation of chorismate (the essential precursor for menaquinone synthesis) are missing.

A reconstitution of menaquinone biosynthesis by transferring the four missing genes (from a suitable source) to facilitate respiratory metabolism in *L. reuteri* KUB-AC5 was considered not feasible in the absence of the precursor chorismate. A strategy using respiratory metabolism to reduce oxidative stress and enhance biomass formation was thus not pursued further.

#### Genes involved in oxidative stress

The genome of *L. reuteri* KUB-AC5 contains genes encoding NADH oxidase (*Nox1*, *Nox2*) and eight possible genes involved in the thioredoxin system (Table 3). Genes encoding ROS scavenging enzyme (*kat*, *sod*) are not present in the genome of *L. reuteri* KUB-AC5.

### Construction of strains expressing genes for ROS scavenging enzymes

The two ROS scavenging enzymes (catalase, superoxide dismutase) were successfully inserted into pSI411 expression plasmids, resulting in two expression plasmids (pSIP411 + *kat* and pSIP411 + *sod*). All constructs were transformed into an *E. coli* intermediate host with the correct gene integration into the vector.

The expression plasmids pSIP411 + *kat* and pSIP411 + *sod* were transformed into *L. reuteri* KUB-AC5 competent cells with a transformation efficiency of 2.2 × 10^–2^ transformants/μg DNA and 5.26 transformants/μg DNA, respectively. The presence of the inserted genes was verified by colony PCR, and colonies containing inserts of the expected size of *kat* (843 bp) and *sod* (630 bp) respectively were selected and designated *L. reuteri* pSIP411 + *kat* and *L. reuteri* pSIP411 + *sod*.

### Expression of ROS scavenging enzymes

**Fig. 3:**
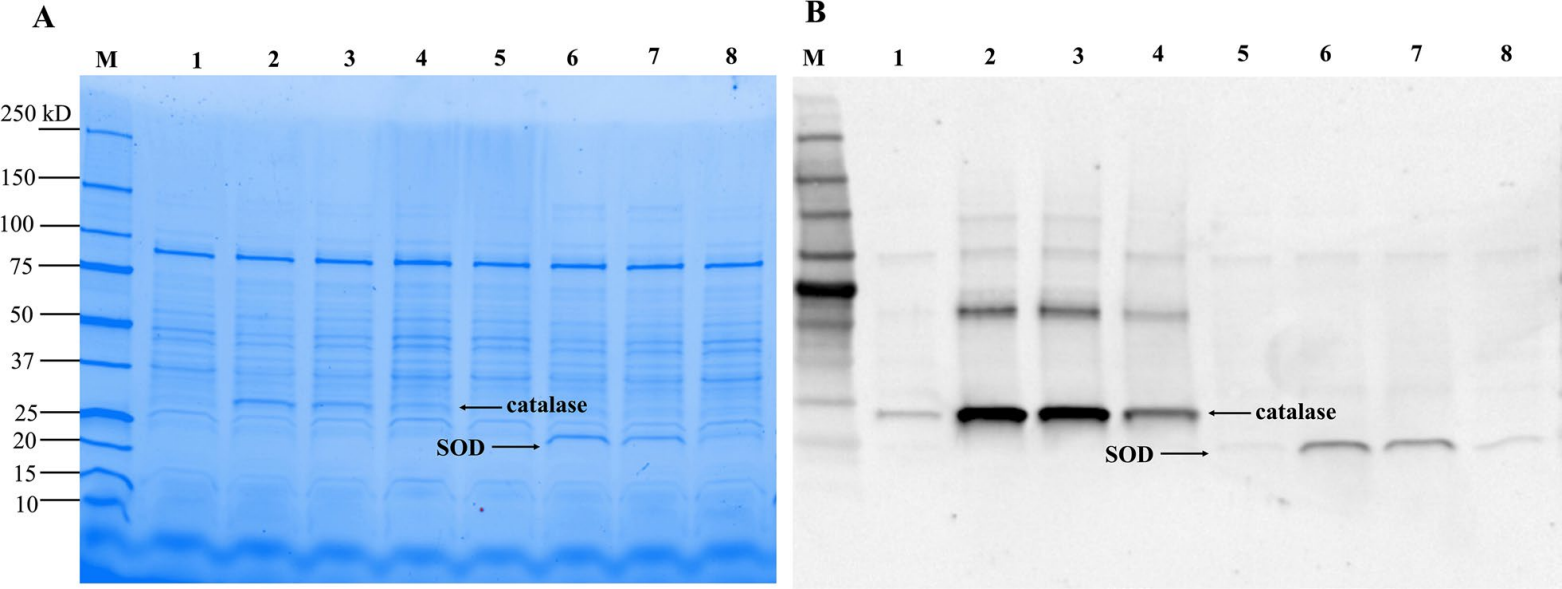
SDS-PAGE **A** and Western blot **B** analysis of recombinant *L. reuteri* pSIP411 + *kat* (lane 1–4) and *L. reuteri* pSIP411 + *sod* (lane 5–8), Lane 1, 5: non-induced at 30 ℃; Lane 2, 6: expressed at 25 ℃; Lane 3, 7: expressed at 30 ℃; Lane 4, 8: expressed at 37 ℃; Lane M: protein marker

The *L. reuteri* pSIP411 + *kat* and *L. reuteri* pSIP411 + *sod* were studied for catalase and superoxide dismutase expression at three different temperature. SDS-PAGE analysis revealed that the induction of *L. reuteri* pSIP411 + *kat* strains at 25 ℃ and 30 ℃ resulted in a more intense protein band at 31 kDa, which corresponds to the predicted size of Mn-catalase (Fig. 3A). In contrast, induction at 37 ℃ results in a faint protein band at the corresponding position (Fig. 3A) and Western blot analysis confirmed a higher catalase enzyme expression at 25 ℃ and 30 ℃ (Fig. 3B). No band corresponding to catalase was detected in non-induced sample on SDS-PAGE but a faint protein band at the expected position was visible on Western blots.

A similar result was obtained for *L. reuteri* pSIP411 + *sod* expression. A band of the expected size of Mn-SOD (24 kDa) was detected on SDS-PAGE in all induced samples. The most favorable temperatures for SOD expression in this strain were 25 ℃ and 30 ℃, whereas at 37 ℃ the band is barely visible (Fig. 3). A very low target protein concentration was detected at 37 ℃ Western blots (Fig. 3B).

Both strains showed high catalase or SOD expression upon induction at 25 ℃ and 30 ℃. Since cultivation and induction at 25 ℃ results in lower biomass formation compared to 30 ℃ and 37 ℃ (Additional file 1), a temperature of 30 ℃ showing both high expression and biomass formation was chosen for further study.

### Aerobic growth phenotype of *L. reuteri* variants strains

**Fig. 4:**
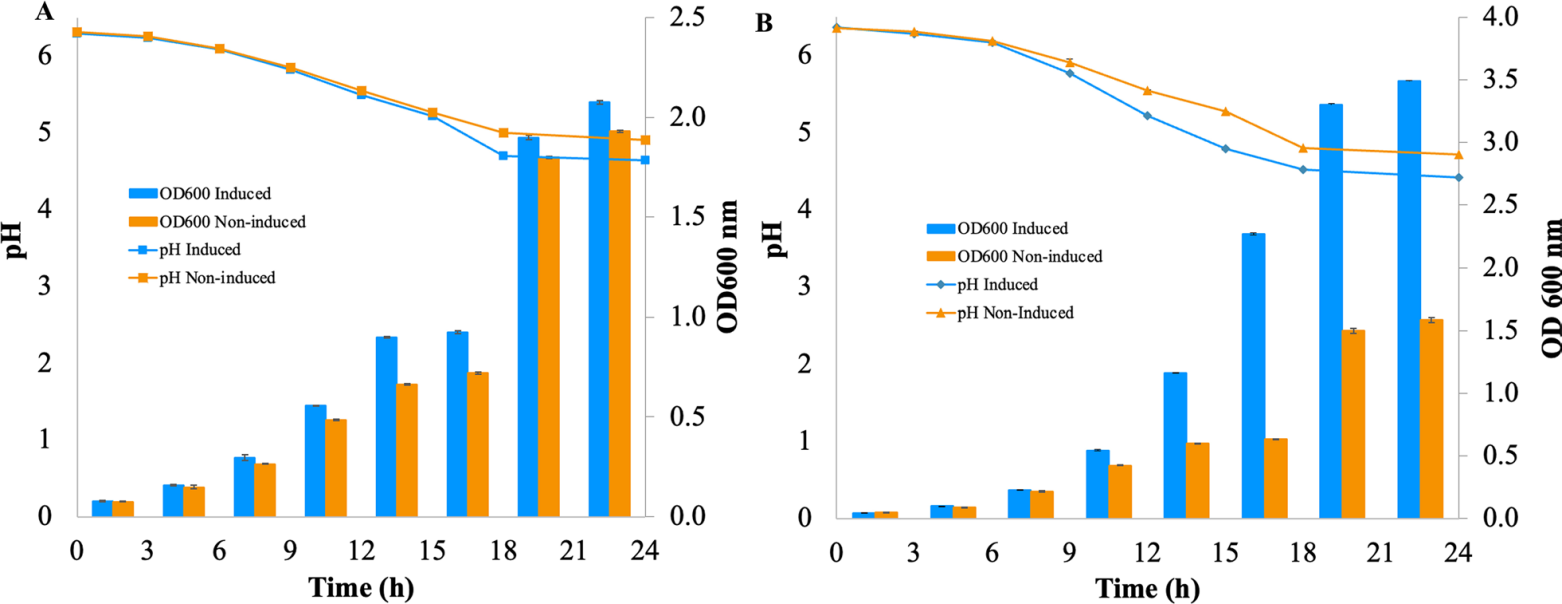
Aerobic growth phenotype (OD 600 nm, pH) of *L. reuteri* pSIP411 + *kat* **A** and *L. reuteri* pSIP411 + *sod* **B** recombinant strains during cultivation at 30 ℃ for 24 h. All results are the mean of three independent experiments; the error bars indicate the standard deviation

*L. reuteri* pSIP411 + *kat* and *L. reuteri* pSIP411 + *sod* recombinant strains were cultivated under aerated (aerobic) and non-aerated (anaerobic) conditions. The growth of *L. reuteri* pSIP411 + *kat* in aerobic cultivation was four times higher (Fig. 4A) than the wild-type strain (Fig. 1) resulted in the maximum biomass concentration at the optical density at 600 nm of 2.0. The same high biomass concentration was also observed in the absence of inducer.

The cultivation of *L. reuteri* pSIP411 + *sod* showed a very high biomass formation at the maximum OD_600_ of 3.5 at 24 h (Fig. 4B). Growth and biomass formation were slightly increased in the absence of inducer.

The pH profile in both recombinant strains was similar with gradually decreasing pH from 6.5 to about 4.5 (Fig. 4).

### Reactive oxygen species and antioxidant enzyme activity measurement in *L. reuteri* KUB-AC5 and its variant strains.

Formation of ROS in *L. reuteri* cells was investigated in the wild-type and the recombinant strains under aerobic and anaerobic conditions. The wild-type strain showed a high fluorescence intensity of 839,339 RFU, indicating an increased formation of ROS in aerobic cultivation compared to static cultivation, where 711,843 RFU were measured (Table 4). In both recombinant strains, low formation of ROS was detected by the reduction of DFC fluorescence intensity in all induced sample. Reduced formation of ROS was also observed in non-induced *L. reuteri* pSIP411 + *kat*, which showed a catalase activity of 432 U/ml without induction and 873 U/ml with induction (Table 4). The lowest ROS formation was detected in the *L. reuteri* pSIP411 + *sod* strain with high SOD activity of 1,213 U/ml, but a reduction was also observed in the absence of inducer, where a SOD activity of 855 U/ml was measured.

**Table 4:** ROS measurement and catalase and SOD activity in *L. reuteri* cells during aerobic and anaerobic condition

Strains and condition	ROS measurement (RFU)	Catalase activity (U/ml)	SOD activity (U/ml)
KUB-AC5 (static)	712 ± 25,0	0	0
KUB-AC5 (150 rpm)	840 ± 7,42	12.9 ± 0.08	1.73 ± 0.02
pSIP411 + *kat* non-induced (150 rpm)	764 ± 7,86	432.6 ± 13.1	7.68 ± 0.55
pSIP411 + *kat* induced (150 rpm)	745,4 ± 4,08	873.4 ± 6.71	9.35 ± 0.51
pSIP411 + *sod* non-induced (150 rpm)	833,6 ± 4,13	34.2 ± 0.11	854.6 ± 12.53
pSIP411 + *sod* induced (150 rpm)	701,8 ± 4,89	47.6 ± 0.41	1,213 ± 31.2

## Discussion

LAB are facultative anaerobic bacteria and are sensitive to oxidative stress to varying degrees. The ability for aerobic growth varies significantly, is highly species-dependent and relies on various mechanisms. The growth profile of *L. reuteri* KUB-AC5 exhibited a strong growth limitation under aerobic cultivation conditions, similar to heterofermentative lactic acid bacteria such as *Limosilactobacillus fermentum* DSM20052 and *Limosilactobacillus reuteri* LMG92113 [50]. We demonstrate here the growth limitation of *L. reuteri* KUB-AC5 under aerobic conditions is caused by higher formation of Reactive oxygen species (ROS). *L. reuteri* KUB-AC5 contains a gene encoding NADH oxidase (*nox*). This enzyme is responsible in many LAB for the removal of oxygen in aerobic conditions to maintain the intracellular redox balance [46], but leads to formation of ROS that are toxic to the cell (Maresca, Zotta, and Mauriello 2018; [5, 14, 16]. To overcome this ROS toxicity, some LAB synthesize Mn-SOD, a ROS scavenging enzyme to detoxify the O_2_^−^ molecule [3, 4, 10]. Enzymes capable of scavenging H_2_O_2_ such as catalase are rare in LAB [16]. In *L. reuteri* KUB-AC5, neither Mn-SOD nor catalase-encoding genes are present in the genome, resulting in the strong growth limitation under aerobic conditions.

Some LAB possess a complete respiratory chain including the biosynthesis of menaquinone as electron shuttle and can switch to respiratory metabolism in the presence of heme, which always needs to be supplemented as there is no heme biosynthesis in LAB [15, 25, 49]. Genomic analysis of *L. reuteri* KUB-AC5 revealed that both NADH dehydrogenase and cytochrome-bd oxidase are present, but the menaquinone biosynthesis pathway is incomplete and lacks four genes [8, 9]. Respiratory metabolism should therefore be functional with supplementation of both heme and menaquinone. A reconstitution of the complete pathway as shown for *Lactiplantibacillus plantarum*, which also lacks four genes [49] appears feasible in principle, but would still require supplementation with chorismate, as the biosynthesis pathway for this precursor is not present. This approach was thus not pursued.

This work therefore focused on a strategy for oxidative stress reduction based on the expression of genes encoding ROS scavenging enzymes, Mn-SOD and Mn-catalase. Two plasmids for inducible expression in Lactobacilli, pSIP409 (data not shown) and pSIP411 were used as backbone, but only pSIP411 could be efficiently transformed into *L. reuteri* KUB-AC5. Both plasmids are the inducible plasmid-system based on the regulatory genes and promoters involved in class II non-lantibiotic bacteriocin production from *L. sakei* sakacin P (*spp* gene cluster) [11, 19]. The three-component system regulates the bacteriocin production, consisting of a secreted peptide pheromone (IP-673), which interacts with a cognate membraneembedded histidine protein kinase (HPK) and transduces the inducer signal from outside of the cell into the cytoplasm. The response regulator (RR) is then activated by transferring a phosphate group from a conserved histidine residue of HPK to an aspartate residue of RR [18], leading to the induction of pSIP409 and pSIP411 promoters [11]. The set of HPK and RR is a two-component regulatory system [18] consisting of two operons, one for target gene expression and another one for the two-component regulatory system controlled by a different promoter [30]. The pSIP409 vector carries the *256rep* origin of replication from *L. plantarum* and has been reported as a narrow-host-range replicon [22, 44, 45], which is limited to several LAB hosts including *L. sakei*, *Lentilactobacillus curvatus* and *L. plantarum* [44, 45]. The unsuccessful transformation of pSIP409 in *L. reuteri* KUB-AC5 suggest that this strain lacks a replication initiation factor for *p256* [44, 45]. In contrast, high transformation efficiency was seen with the constructs based on pSIP411, which harbors an *SH71rep* origin of replication. In contrast to 256rep, *SH71rep* is a high copy number replicon from *L. lactis* [35] that is reported as a broad-host-range replicon and is also functional in *L. reuteri* DSM20016 and *L. reuteri* ATCC PTA 6475 [22] as well as *L. reuteri* KUB-AC5.

Expression of both Mn-catalase and Mn-SOD showed high activities when cultivation and induction were performed at 25 ℃ and 30 ℃, but not at 37 ℃. This result is in contrast to previously reported results of β-galactosidase expression in *L. plantarum* using the pSIP system, where higher protein expression was observed at 37 ℃ and was attributed to a higher growth rate at 37 ℃ [33]. In *L. reuteri* KUB-AC5, lower cell density was reached at 25 ℃, but at 30 ℃ and 37 ℃ the cell density after 24 h cultivation was similar. The higher protein expression at low temperature (25 ℃ and 30 ℃) could be due to higher solubility of the heterologous protein [47]. Lowering the induction temperature slows down cell proliferation processes including the rate of transcription and translation, which results in lower proportions of mis-folded protein and minimizes protein aggregation [41]. A higher protein expression at 30 ℃ was also reported for *L. reuteri* DSM 20016 with a pTRKH3-*erm*GFP expression plasmid [35].

Catalase activity and ROS reduction were measured in both induced and non-induced *L. reuteri* pSIP411 + *kat* cultures (Table 4) and confirmed by Western blot, suggesting a leaky promoter driving a certain level of transcription in the absence of inducer. The leakage of the Sakacin-inducible promoter has been previously reported [23, 33]. Non-induced cultures also showed an only mildly slower growth behavior compared to induced cultures, and both non-induced and induced cultures grew to cell densities as observed for non-aerated conditions. *L. reuteri* KUB-AC5 possesses a Thioredoxin system, which is a crucial protective system against H_2_O_2_ using disulfide reductase activity regulating protein dithiol/disulfide balance [26, 27, 37]. The results presented here suggest that, together with the Thioredoxin system, the catalase activity resulting from the leaky promoter is largely sufficient for the reduction of oxidative stress caused by hydrogen peroxide. It is also noteworthy that the reduction in total ROS activity in catalase-expressing cells is not entirely proportional to the increased biomass formation: biomass formation is restored to the levels observed in non-aerated cultures, even though the total ROS activity remains slightly elevated. *L. reuteri* pSIP411 + *sod* cultures showed a significantly higher biomass formation, beyond the levels of non-aerated cultures, and strong reduction of ROS activity to levels comparable with anaerobic conditions. This suggests that the impact on growth limitation caused by superoxide anion radicals is more pronounced than that of hydrogen peroxide (or other forms of ROS). SOD activity decomposes superoxide anions to H_2_O_2_, which is still toxic to the cells. Our results suggest that elimination of the superoxide anion radicals is a major factor in counteracting detrimental effects of ROS, and that the resulting formation of hydrogen peroxide can be handled by the native Thioredoxin system. Whether a co-expression of both Mn-SOD and Mn-catalase can further reduce total ROS activity (to levels even below those of non-aerated cultures), and whether this has additional impact on biomass formation will be the subject of further studies.

## Supplementary Information

**Additional file 1:** The cell growth of Limosilactobacillus reuteri variant strains in aerobic condition at different temperature.

## Data Availability

All data generated or analyzed during this study are included in this published article [and its additional files].
