# Supplementary material for: Development of high cell density Limosilactobacillus reuteri KUB-AC5 for cell factory using oxidative stress reduction approach
Source: Microb Cell Fact. 2023 Apr 29;22:86. doi: 10.1186/s12934-023-02076-4 (PMC10149017; doi:10.1186/s12934-023-02076-4)
Supplement: Supplementary file 1 — Additional file 1: The cell growth of Limosilactobacillus reuteri variant strains in aerobic condition at different temperature. [file 12934_2023_2076_MOESM1_ESM.docx]

**Additional file 1**

The cell growth of *Limosilactobacillus reuteri* variant strains in aerobic condition at different temperature

| Strains | OD 600 nm at different cultivation temperature | | |
| --- | --- | --- | --- |
|  | 25°C | 30°C | 37°C |
| *L. reuteri* pSIP411+*kat* | 1.85±0.05 | 2.40±0.20 | 2.45±0.11 |
| *L. reuteri* pSIP411+*sod* | 1.93±0.16 | 3.35±0.13 | 3.46±0.10 |
